# Resveratrol Modulation of Protein Expression in *parkin*-Mutant Human Skin Fibroblasts: A Proteomic Approach

**DOI:** 10.1155/2017/2198243

**Published:** 2017-09-12

**Authors:** Daniele Vergara, Antonio Gaballo, Anna Signorile, Anna Ferretta, Paola Tanzarella, Consiglia Pacelli, Marco Di Paola, Tiziana Cocco, Michele Maffia

**Affiliations:** ^1^Department of Biological and Environmental Sciences and Technologies, University of Salento, Lecce, Italy; ^2^Laboratory of Clinical Proteomic, “Giovanni Paolo II” Hospital, ASL-Lecce, Lecce, Italy; ^3^CNR NANOTEC-Istituto di Nanotecnologia, Polo di Nanotecnologia, Campus Ecotekne, Lecce, Italy; ^4^Department of Basic Medical Sciences, Neurosciences and Sense Organs, University ‘A. Moro', Bari, Italy; ^5^Department of Clinical and Experimental Medicine, University of Foggia, Foggia, Italy; ^6^Institute of Clinical Physiology, National Research Council, Campus Ecotekne, Lecce, Italy

## Abstract

In this study, we investigated by two-dimensional gel electrophoresis (2-DE) and mass spectrometry (MS) analysis the effects of resveratrol treatment on skin primary fibroblasts from a healthy subject and from a *parkin*-mutant early onset Parkinson's disease patient. Parkin, an E3 ubiquitin ligase, is the most frequently mutated gene in hereditary Parkinson's disease. Functional alteration of parkin leads to impairment of the ubiquitin-proteasome system, resulting in the accumulation of misfolded or aggregated proteins accountable for the neurodegenerative process. The identification of proteins differentially expressed revealed that resveratrol treatment can act on deregulated specific biological process and molecular function such as cellular redox balance and protein homeostasis. In particular, resveratrol was highly effective at restoring the heat-shock protein network and the protein degradation systems. Moreover, resveratrol treatment led to a significant increase in GSH level, reduction of GSSG/GSH ratio, and decrease of reduced free thiol content in patient cells compared to normal fibroblasts. Thus, our findings provide an experimental evidence of the beneficial effects by which resveratrol could contribute to preserve the cellular homeostasis in *parkin*-mutant fibroblasts.

## 1. Introduction

Parkinson's disease (PD) is a multifactorial neurodegenerative disorder that predominantly affects the population over 65 years of age [1]. From a clinical point of view, the disease is characterized by the presence of motor deficit associated with abnormal intracellular protein deposits called Lewy bodies (LBs) and loss of dopaminergic neurons, primarily, within the *substantia nigra pars compacta* (SNpc) [2]. Several risk factors were identified including disease-causing mutations in a specific set of genes that mediate the autosomal-dominant or autosomal-recessive forms of PD [3], among which mutations in *alpha synuclein* (SNCA) and in *leucine-rich repeat kinase 2* (LRRK2) are responsible for autosomal-dominant PD forms whereas mutations in *parkin*, *PTEN-induced putative kinase 1* (PINK1), *DJ-1*, and *ATP13A2* are accountable for PD that displays an autosomal recessive mode of inheritance [3].

The most common mutant gene implicated in familial PD is *parkin*, and various loss-of-function mutations occurring in both alleles produce an aggressive, generally early form of PD [4–6]. Parkin is a cytosolic protein with E3 ubiquitin ligase activity, for ubiquitin-proteasome-dependent protein turnover, with a central role in mitochondrial maintenance and turnover. In response to mitochondrial damage, PINK1 induces the activation of parkin by phosphorylation. Once activated, parkin conjugates ubiquitin onto proteins on the outer mitochondrial membrane (OMM), leading to mitochondrial engulfment by the autophagosome via the endosomal sorting complexes required for transport (ESCRT) machinery [7–9]. Pathogenic mutations of *parkin* lead to the accumulation of damaged mitochondria and are associated with several cellular dysfunctions including impaired energy metabolism, deregulated reactive oxygen species (ROS) production, failure of ubiquitin-proteasome pathway, and protein misfolding [10–13].

Mass spectrometry- (MS-) based studies made possible to shed lights on the cellular pathways modified after parkin loss [14–16]. Proteomic analysis of human primary fibroblasts isolated from patients with a genetic deficit of *parkin* revealed that parkin is implicated in the modulation of multiple cellular functions including cytoskeleton structure dynamics, calcium homeostasis, oxidative stress response, and protein and RNA processing [17]. In this cellular model, the absence of parkin has also been associated with a specific phospholipid and glycosphingolipid lipidomic profile likely related to dysfunction of autophagy and mitochondrial turnover [18].

Current pharmacological treatments of PD remain largely symptomatic, and the development of new therapeutic strategies may provide effective alternative treatment options. In recent years, resveratrol has emerged as a compound conferring protective effects against metabolic and other stresses in age-related diseases, including neurodegeneration [19]. Resveratrol (*trans*-3,5,4′-trihydroxystilbene) a dietary polyphenol present in several medical plants [20] demonstrated multiple biological activities, including anti-inflammatory properties [21], antioxidant effects [22], and neuroprotection in both cerebral ischemia and neurodegenerative diseases, such as Alzheimer's disease and Parkinson's disease [23, 24]. Studies performed on animal models of PD have shown that resveratrol protects dopaminergic neurons from 6-hydroxydopamine- (6-OHDA-) and 1-methyl-4-phenyl-1,2,3,6-tetrahydropyridine- (MPTP-) induced degeneration, possibly via modulation of autophagy and proinflammatory pathways [25–27]. Ex vivo models of PD also gained interest for the preclinical assessment of the biological and medical properties of resveratrol. Previous studies of our group have shown that resveratrol treatment of *parkin*-null cellular model induced a partial rescue of mitochondrial functions and oxidative stress through the activation of the AMP-activated protein kinase (AMPK)/sirtuin 1 (SIRT1)/peroxisome proliferator-activated receptor gamma coactivator 1-alpha (PGC-1*α*) pathway [28].

In this work, we investigated by two-dimensional gel electrophoresis (2-DE) and mass spectrometry (MS) analysis the effects of resveratrol in *parkin*-mutant human skin fibroblasts. The analysis of proteins differentially expressed revealed that resveratrol treatment acts on deregulated specific biological process and molecular function such as cellular redox balance and protein homeostasis. In particular, resveratrol was highly effective at restoring the heat-shock protein network and the protein degradation systems as well as the GSH/GSSG ratio, together responsible for the maintaining of the normal protein homeostasis which is essential to proper cellular function.

## 2. Materials and Methods

### 2.1. Cell Culture Conditions

Primary skin fibroblasts from one subject affected by an early onset PD with *parkin* compound heterozygous mutations (P1 with del exon2-3/del exon3) and from the parental healthy subject (CTR) [13, 28] were obtained by explants from skin punch biopsy, after informed consent. Cells were grown in high-glucose Dulbecco's modified Eagle's medium (DMEM) supplemented with 10% (*v*/*v*) fetal bovine serum (FBS), 1% (*v*/*v*) L-glutamine, and 1% (*v*/*v*) penicillin/streptomycin, at 37°C in a humidified atmosphere of 5% CO_2_.

In cell culture experiments, resveratrol (Sigma, R5010) was dissolved in dimethyl sulfoxide (DMSO) and used at the concentration of 25 *μ*M; control cells were treated with an equivalent volume of DMSO (vehicle, 0.02%).

### 2.2. Sample Preparation and Protein Separation by 2-DE

Cell pellets were dissolved in a lysis solution that contained 7 M urea, 2 M thiourea, 4% CHAPS, and a cocktail of protease and phosphatase inhibitors (Biotool). Samples were then sonicated on ice for three rounds of 10 s and processed according to the methods described before [29–31] with minor modification. Briefly, total proteins (80 *μ*g) were diluted up to 250 *μ*L with a rehydration buffer (7 M urea, 2 thiourea, 4% CHAPS, 65 mM DTT, and 0.5% *v*/*v* IPG buffer) and applied to IPG strips (13 cm, pH 3–10 NL). IEF and second dimension were carried out using an IPGphor IEF and a Hoefer SE 600 Ruby electrophoresis system (GE Healthcare). The IPG strips were loaded and run on a 12% SDS-PAGE gel and stained according to the protocol of Chevallet et al. [32]. Gels were scanned by Image Master scanner and analyzed by Image Master software 5.0 (GE Healthcare) using TIF format images at 300 dpi. Spot detection and matching were carried out by the software tools and corrected manually when necessary. The parameter that we used to compare gels was the volume % (vol %) of each spot, expressed as percentage of the spot volume over the total volume of all spots in the gel. Student's *t*-test with a set value of *p* < 0.05 was used to determine significant differences in protein expression levels. Each experiment was performed three times independently.

### 2.3. Mass Spectrometry Identification and Data Analysis

#### 2.3.1. Protein Identification by nHPLC ESI-Trap Analysis

Protein spots were manually excised from 2D gels, destained with H_2_O_2_, and subjected to trypsin digestion followed by identification using an nLC-MS/MS as described [29–31]. The nano-HPLC separation of peptides was performed using a Proxeon Easy-nLC (Thermo Fisher Scientific, Waltham, MA, USA) equipped with a NS-AC-10 analytical column, 5 *μ*M, C18, 375 *μ*M OD × 75 *μ*M ID × 10 cm length, protected by an NS-MP-10 guard column, 5 *μ*M, C18, 375 *μ*M OD × 100 *μ*M ID × 2 cm length (Nano Separations, Nieuwkoop, The Netherlands).

#### 2.3.2. Protein Identification by MALDI-TOF/TOF

Spots of interest were dehydrated with 50 *μ*L of acetonitrile and trypsin digested overnight as described [29]. Resulting peptides were then concentrated by using C18 Zip-Tips (Millipore) and eluted with 2 *μ*L of CHCA matrix (66 *μ*L TFA, 0.1%; 33 *μ*L ACN) directly on an MTP AnchorChipTM 384 BC plate (Bruker Daltonics). Peptides were analyzed by peptide mass fingerprinting (PMF) and MS/MS analysis with a MALDI-TOF/TOF Ultraflextreme (Bruker Daltonics) in positive ion reflector mode (m/z range 500–4000), operating at 1 kHz frequency and controlled by the FlexControl 3.4 software. External calibration was performed using the Peptide Standard Calibration II (Bruker Daltonics). Spectra were processed using the software FlexAnalysis (version 3.4, Bruker Daltonics) and precursor ions with a signal to noise ratio greater than 10 selected for subsequent MS/MS analysis.

Compound lists were submitted to Mascot using the software BioTools (version 3.2, Bruker Daltonics). Peptide masses were compared with those present in the Swiss-Prot human protein database. Database search was performed using the following parameters: peptide tolerance, 0.05 Da; fragment mass tolerance, 0.25 Da; enzyme, trypsin; missed cleavage, one; and instrument, MALDI-TOF/TOF. Peptide tolerance was set to ±1.2 Da, the MS/MS tolerance was set to 0.6 Da, and searching peptide charges were of 1+, 2+, and 3+ for ESI-Trap data. Moreover, carbamidomethyl (C) and oxidation (M) were chosen as fixed and variable modifications, respectively. Identified proteins were subjected to Gene Ontology (GO) analysis and protein-protein interaction (PPI) analysis by STRING software (version 10.0, http://string-db.org/).

### 2.4. GSH and GSSG Determination

For GSH and GSSG assay, fibroblasts were collected by trypsinization and centrifuged at 500 ×g and then resuspended in cold 5% (*w*/*v*) metaphosphoric acid. The sample was exposed to ultrasound energy for 15 s at 0°C and centrifuged at 12,000 ×g for 5 minutes. The supernatant was used to determine GSH and GSSG concentration using an enzymatic/colorimetric assay kit (Enzo Life Sciences) according to the manufacturer's instructions. The measurements were performed on a Victor 2030 Explorer (PerkinElmer). Total protein concentration was determined by Bio-Rad protein assay. GSH and GSSG levels were normalized to protein concentration and expressed as nmol/mg protein.

### 2.5. P-SH Measurement

Cells were collected by trypsinization and centrifugation at 500 ×g and then resuspended in phosphate-buffered saline (PBS), pH 7.4, in the presence of the protease inhibitor phenylmethanesulfonyl fluoride (PMSF). The content of P-SH in total cellular lysate was measured with a modification of the Ellman's procedure [33]. The protein pellet was obtained by precipitation with 4% SSA and centrifugation. Next, the pellet was resuspended in 6 M guanidine, pH 6.0. Optical density was read spectrophotometrically at 412 and 530 nm before and after 30 min of incubation with 10 mM 5,5-dithiobis (2-nitrobenzoic acid). P-SH concentrations were calculated using a standard curve generated with reduced glutathione.

### 2.6. Analysis of Glutathionylated Proteins

Glutathionylated proteins were detected by Western blot analysis of cellular lysates after nonreducing SDS-PAGE. Cells were collected by trypsinization and centrifugation at 500 ×g and then resuspended in PBS, pH 7.4, containing the protease inhibitor PMSF and supplemented with 5 mM *N*-ethylmaleimide (NEM) to block unreacted thiol group. Total cellular proteins (50 *μ*g per lane) were separated on 12% (*w*/*v*) SDS-PAGE and transferred to nitrocellulose membranes. Glutathionylated proteins were visualized with anti-GSH antibody (1 : 1000, Thermo Fisher Scientific number MA1-7620). Glyceraldehyde-3-phosphate dehydrogenase (GAPDH) (Sigma) was used as loading control. After several washes in Tween/Tris-buffered saline solution (TTBS), the membrane was incubated for 60 minutes with an anti-rabbit or anti-mouse IgG peroxidase-conjugate antibody (diluted 1 : 5000). Immunodetection was then performed with the enhanced chemiluminescence (ECL) (Bio-Rad, Milan, Italy). The VersaDoc imaging system was used to perform densitometric analysis (Bio-Rad, Milan, Italy).

### 2.7. Western Blot Analysis

Whole proteins were extracted with RIPA buffer (Cell Signaling) and quantified by the Bradford protein assay (Bio-Rad). Samples were separated by 10% SDS-PAGE and transferred to the Hybond ECL nitrocellulose membrane. The membranes were blocked overnight in Blotto A (Santa Cruz) at 4°C and subsequently probed by the appropriately diluted primary antibodies for 2 h at room temperature. Protein bands were visualized by incubating with a horseradish peroxidase-conjugated secondary antibody (Amersham, ECL Western blotting detection reagents).

## 3. Results and Discussion

### 3.1. Proteomic Profile Alteration in PD Fibroblasts

**Figure 1:**
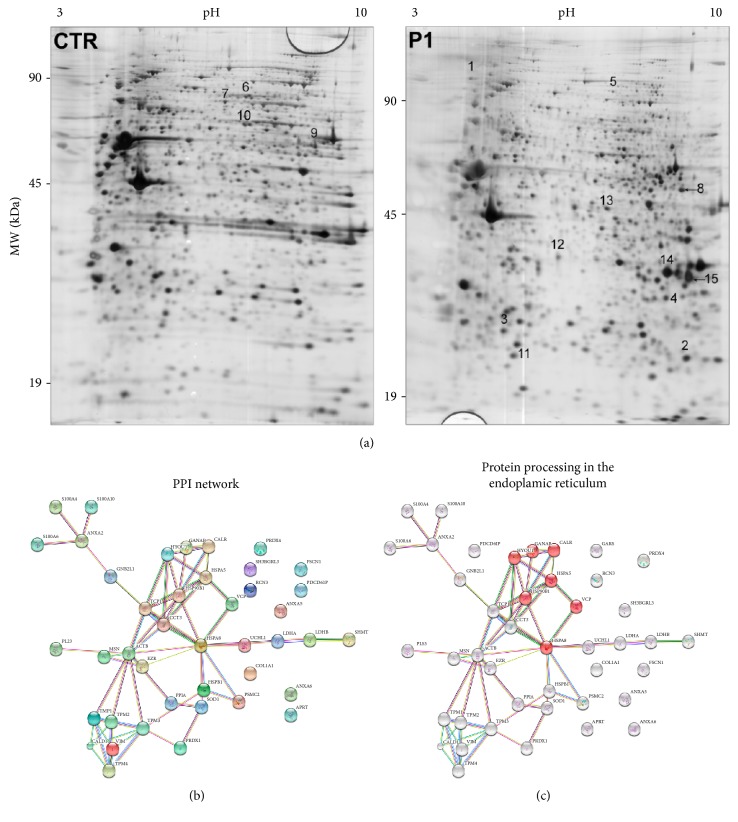
Representative 2-DE gel map of proteins isolated from human CTR and P1 fibroblasts. (a) A total of 80 *μ*g of proteins were separated by 2-DE using a 13 cm IPG strip pH 3–10 NL and 12% SDS-PAGE. Proteins were visualized by silver staining. Spot numbers indicate proteins that were differentially regulated between CTR and P1 samples. (b, c) Bioinformatics analysis of differentially expressed proteins. (b) A high confidence protein-protein interaction network generated with STRING using our protein dataset is shown. The network nodes are input proteins. The edges represent the predicted functional associations. An edge may be drawn with up to 7 differently colored lines—these lines represent the existence of the seven types of evidence used in predicting the associations. A red line indicates the presence of fusion evidence; a green line, neighborhood evidence; a blue line, coocurrence evidence; a purple line, experimental evidence; a yellow line, textmining evidence; a light blue line, database evidence; and a black line, coexpression evidence. (c) Proteins involved in protein processing in the endoplasmic reticulum proteins are highlighted in red (HYOU1, GANAB, CALR, HSPA5, HSP90B1, VCP, and HSPA8) in the main PPI network.

In our previous work, we analyzed, by 2-DE and MALDI-MS, proteins isolated from fibroblast cultures of healthy subjects and patients affected by PD [17]. This comparative proteomic approach led to the identification of several differentially expressed proteins. Here, we modified some of the experimental parameters used previously to separate proteins from fibroblast cultures, including 2-DE buffer composition and isoelectric focusing conditions, in order to increase the number of proteins separated by 2-DE and the potential number of differentially expressed proteins identified after comparative analysis. We focused on control (CTR) and PD patient (P1) fibroblasts that we recently characterized for a variety of cellular alterations associated with the modulation of metabolic and cytoskeletal proteins [13, 34]. With these technical improvements, we identified 15 additional differentially expressed proteins which are not yet identified in the previous work [17]. The identity of these proteins was determined by MALDI-TOF MS/MS and listed in Table 1. By combining these new results with the precedent group of identified proteins, we obtained a dataset of 44 distinct and well-annotated differentially expressed proteins that were subjected to bioinformatics analysis. GO classification and protein-protein interaction network (PPI) of this dataset are shown in Tables 2, 3, and 4 and Figure 1(b). Data showed a significant decrease, in P1 compared to CTR cells, of the expression of protein related to several biological processes like those involved in cell movement or subcellular components as well as those involved in regulating, assembly and protein folding, calcium ion binding, and unfolded protein binding. Kyoto Encyclopedia of Genes and Genomes (KEGG) pathway enrichment analysis identified protein processing in the endoplasmic reticulum (ER) as significantly modified (Table 4). This includes a list of 7 well-connected proteins HYOU1, GANAB, CALR, HSPA5, HSP90B1, VCP, and HSPA8 as determined by PPI analysis (Figure 1(c)). Most of these proteins belong to the heat-shock protein (HSP) family, and all of them participate in protein folding. HSPs have become a research focus in PD because the pathogenesis of this disease is highlighted by the intracellular protein misfolding and inclusion body formation. HSPs are mainly involved, by interaction with different cochaperones, in folding nascent polypeptides to their appropriate conformation and refolding mild denatured/damaged proteins. Moreover, working together with the ubiquitin-proteasome system (UPS), they are involved in the decomposition of aberrant proteins. In addition, HSPs may possess antiapoptotic effects and keep the cellular homeostasis against stress conditions [35–38]. Evidence involving a direct role for UPS in PD results from the association between genetic mutations in *parkin* with familial parkinsonism [4].

It is noteworthy to highlight the high level of the ubiquitin carboxyl-terminal hydrolase isozyme L1 (UCHL1), a protein component of UPS, observed in PD fibroblasts (3.8-fold increase with respect to CTR cells) (Table 1). In addition to its major function related to protein degradation as a component of UPS [39], UCHL1 possesses an ubiquitin ligase-like enzymatic activity [40], placing it in a pathway potentially related to parkin. It is reported that interaction with parkin promotes UCHL1 lysosomal degradation [41] and consequently the lack of parkin could lead to the abnormal UCHL1 accumulation in PD patient cells. P1 cells are also characterized by a deregulation of redox state, and, according to previous work showing a different expression level of protein involved in oxidative stress response [17], 2-DE data revealed a significant increase of peroxiredoxin-1 (PRDX1) in P1 with respect to CTR cells (Table 1).

A differential expression level of energy metabolism-associated proteins was also observed. L-lactate dehydrogenase A chain (LDH-A) and B chain (LDH-B) resulted both overexpressed in P1 fibroblasts. This is consistent with the finding that P1 cells, characterized by mitochondrial dysfunctions, showed a high glycolytic ATP production, lactate level, and intracellular LDH activity [13].

Perturbation of protein folding homeostasis is a common pathologic feature of many neurodegenerative diseases, including Alzheimer's disease and PD [42, 43]. Protein folding in the ER is finely regulated by various conditions including redox state and calcium concentrations. In the list of the molecular functions (Table 3), we observed that the calcium ion binding and unfolded protein binding pathways were significantly enriched in CTR versus P1 cells suggesting that the defect of these functions in P1 cells could be responsible for the altered cellular homeostasis. Furthermore, according to our previous work [17, 34], we detected in PD samples with respect to CTR a significantly lower level of the protein ezrin, a member of the ERM (ezrin, radixin, and moesin) protein family, involved in the connection of major cytoskeletal structures to the plasma membrane [44].

Overall these new data, together with the previously obtained results, point to the involvement of parkin in the regulation of a complex network of processes related to cytoskeletal rearrangements and protein folding organization in the ER [17, 34].

### 3.2. Establishing a Proteomic Expression Signature Associated with Resveratrol Treatment in Control and PD Fibroblasts

Since *in vitro* and *in vivo* studies demonstrated the promising effects of resveratrol on neuronal diseases, with a well-described effect in retarding or even reversing the accelerated rate of neuronal degeneration [25–27, 45, 46], we went through the study of protein expression profile in a cellular PD model to gain further insights into the molecular effects induced by resveratrol treatment.

**Figure 2:**
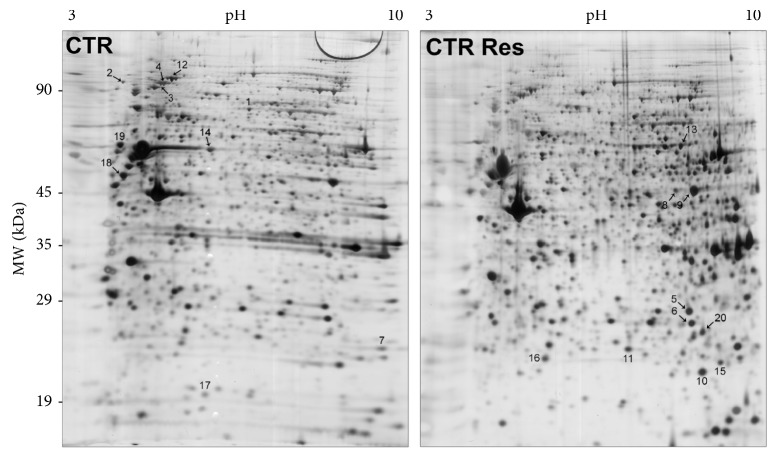
Representative 2-DE gel map of CTR and CTR-Res-treated cell proteins. A total of 80 *μ*g of proteins were separated by 2-DE using a 13 cm IPG strip pH 3–10 NL and 12% SDS-PAGE. Proteins were visualized by silver staining. Spot numbers indicate differentially expressed proteins.

**Figure 3:**
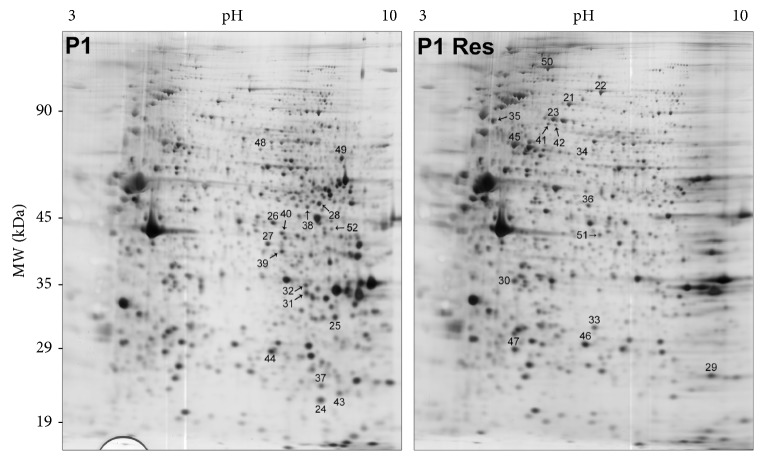
Representative 2-DE gel map of P1 and P1-Res-treated cell proteins. A total of 80 *μ*g of proteins were separated by 2-DE using a 13 cm IPG strip pH 3–10 NL and 12% SDS-PAGE. Proteins were visualized by silver staining. Spot numbers indicate differentially expressed proteins.

**Figure 4:**
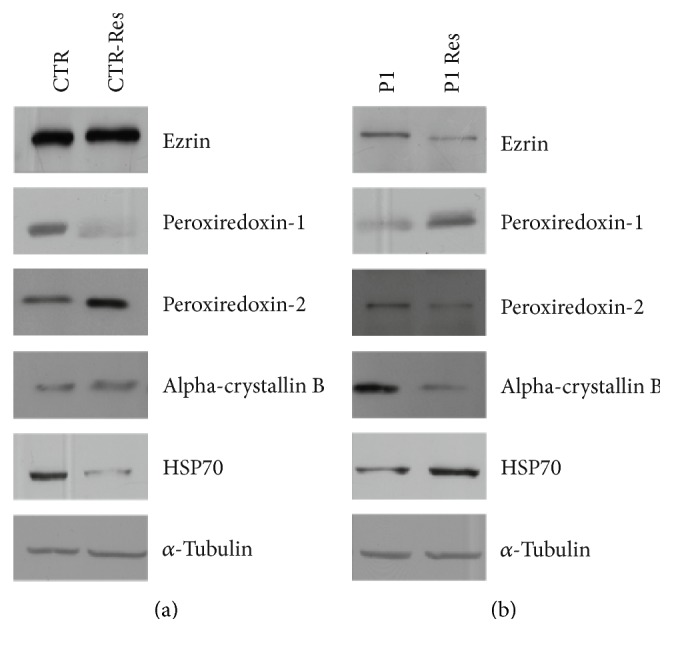
Western blot analysis of CTR and P1 fibroblasts treated with resveratrol. CTR and P1 cells were treated with resveratrol at the concentration of 25 *μ*M for 24 h. Proteins were separated by SDS-PAGE and probed with the following antibodies: ezrin (1 : 2000, Santa Cruz, sc-20773), alpha B crystallin (1 : 1000, Proteintech, 15808-1-AP), peroredoxin-1 (1 : 2000, Santa Cruz, sc-7381), peroredoxin-2 (1 : 2000, Santa Cruz, sc-33572), peroredoxin-6 (1 : 2000, Santa Cruz, sc-393024), and heat-shock protein (HSP70) (1 : 2000, Sigma, H51747). *α*-Tubulin was used as loading control (1 : 4000, Santa Cruz, sc-23948). This image is representative of three independent experiments.

Protein expression was investigated by 2-DE and MALDI-MS/MS analysis after 24 h of treatment with resveratrol at the concentration of 25 *μ*M or with vehicle (DMSO) (Figures 2 and 3). A specific dataset of proteins resulted differentially expressed in CTR and P1 fibroblasts and listed in Tables 5 and 6. These datasets were functionally annotated using the software STRING. The most enriched GO terms of molecular functions and biological processes are depicted in Tables 7, 8, and 9. After resveratrol treatment, CTR fibroblasts showed changes in the protein level associated with peroxiredoxin activity (Table 8). This is in line with previous observations that linked resveratrol action with the modulation of enzymes involved in the ROS metabolism [28, 47]. The effect of resveratrol treatment on proteins with peroxiredoxin activity was also observed, to a lesser extent, in P1 fibroblasts (Table 9). These results were validated by Western blotting analysis (Figure 4()). Consistent with MS data, P1 resveratrol-treated cells expressed higher levels of PXR1 and lower levels of PXR6 compared to untreated cells. By contrast, CTR cells showed a trend towards a lower PXR1 and higher PXR6 expression in resveratrol-treated cells compared to untreated cells.

### 3.3. Modulation of Redox Status of Sulfhydryl Groups upon Treatment with Resveratrol

**Figure 5:**
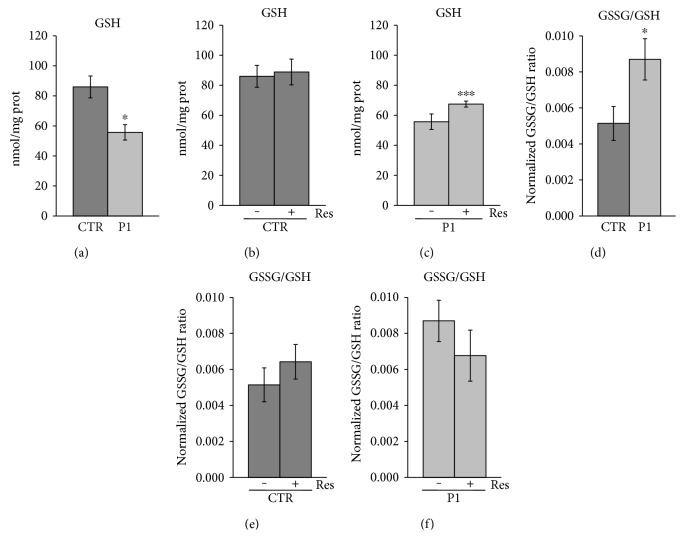
Effect of resveratrol treatment on GSH and GSSG content in patient and control cells. Primary fibroblasts from patient (P1) and control fibroblasts (CTR) were grown as specified in *Materials and Methods*. As indicated, cells were incubated with 25 *μ*M resveratrol for 24 hours (Res). GSH and GSSG content were determined in total cellular lysate. (a) The histogram represents the mean values of GSH basal level ± SEM of different experiments (*n* = 3). (b, c) Effect of Res treatment on GSH content in CTR and P1 cells. The values are means ± SEM of different experiments (*n* = 3). (d) The histogram represents the mean values of normalized GSSG/GSH ratio ± SEM of different experiments (*n* = 3). (e, f) Effect of Res treatment on GSSG/GSH ratio in CTR and P1 cells. The values are means ± SEM of different experiments (*n* = 3). *p* value was determined by Student's *t-*test, ^∗^*p* < 0.05, ^∗∗∗^*p* < 0.001.

Mammalian cells have a well-defined set of antioxidant enzymes, which includes superoxide dismutase, catalase, glutathione peroxidases, and peroxiredoxins, ubiquitous enzymes that have emerged as an important and widespread peroxide and peroxynitrite scavenging system [48]. PD is characterized by changes in oxidative balance, and the loss of glutathione (GSH) level is one of the earliest biochemical changes detectable in PD [49]. GSH is a major component of cellular antioxidant system, whose reduced and oxidized forms (GSH and GSSG) act in concert with other redox-active compounds (e.g., NAD(P)H) to regulate and maintain cellular redox status [50]. Glutathione depletion may occur as a defect of its synthesis, as well as its metabolism, when the redox state of the cells is altered. In these conditions, the GSSG produced can be reduced back to GSH, but the formation and export of GSH conjugates could lead to GSH depletion. As shown in Figure 5, total GSH level was significantly lower in P1 fibroblasts as compared to CTR cells (Figure 5(a)). However, treatment of P1 cells with 25 *μ*M Res for 24 h resulted in a significant increase of GSH content (Figure 5(c)), whereas the treatment had no effect on the GSH level in CTR cells (Figure 5(b)). The analysis of oxidized glutathione (GSSG) revealed an increase of GSSG/GSH ratio in P1 cells (Figure 5(d)), which was partially reversed after resveratrol treatment, though the value did not reach a statistical significance (*p* = 0.06) (Figure 5(f)). These data are consistent with the observation that resveratrol could act positively on glutathione homeostasis by increasing the activity and the expression, through NRF2, of glutamate cysteine ligase (GCL), the rate-limiting enzyme for de novo GSH synthesis that catalyzes the formation of *γ*-glutamylcysteine [51, 52].

**Figure 6:**
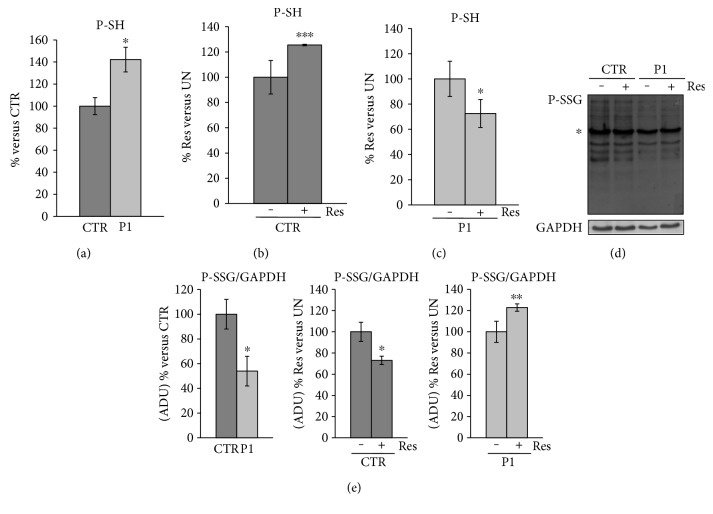
Effect of resveratrol treatment on free thiol groups (P-SH) of protein and glutathionylation in patient and control cells. Primary fibroblasts from patient (P1) and control fibroblasts (CTR) were grown as specified in *Materials and Methods*. As indicated, cells were incubated with 25 *μ*M resveratrol for 24 hours (Res). The P-SH levels were measured in total cellular lysate. (a) The histogram represents the percentage changes with respect to P-SH content of CTR. (b, c) The histograms represent the percentage changes with respect to P-SH content of CTR and P1 untreated cells (UN). (a, b, c) The values are means ± SEM of different experiments (*n* = 3). (d) A representative image of glutathionylated proteins. Proteins of cellular lysate were loaded on 12% SDS-PAGE, under nonreducing conditions, transferred to nitrocellulose membranes, and immunoblotted with the antibody against GSH (P-SSG). Protein loading was assessed with the GAPDH antibody. (e) Densitometric analysis of PSSG proteins, normalized to GAPDH, was performed considering all bands immune-revealed by the GSH antibody, excluding the protein band marked with an asterisk. This band is not specific; it also appears in SDS-PAGE under reducing conditions (data not shown). The histograms represent the percentage changes of ADU express as P-SSG/GAPDH ratio. The values are means ± SEM of different experiments (*n* = 3). *p* value determined by Student's *t*-test, ^∗^*p* < 0.05, ^∗∗^*p* < 0.01, ^∗∗∗^*p* < 0.001.

Changes in GSH metabolism prompted us to investigate the redox state of thiol groups of protein (P-SH). The thiol groups of the protein are characterized by a reversible formation of a mixed disulfide bond between two cysteines and with glutathione (glutathionylation), which controls correct protein folding and represents an emergent mechanism of posttranslational modification to regulate signal transduction [53]. Quantitative analysis of free sulfhydryl groups of protein (P-SH) in total cellular lysate reveals higher levels of P-SH in P1 cells respect to CTR cells (Figure 6(a)), and this result could reflect the high steady-state cellular redox state (NADH/NAD^+^) measured in these cells [28]. As expected, resveratrol treatment of CTR cells results in a significant increase of P-SH (Figure 6(b)), reflecting the antioxidant capacity of the employed polyphenol [54]. The P-SH increase could be potentially related to the decreased level of two disulfide isomerases, PDIA3 and P4HB, as detected by the proteomic analysis (Table 6). These proteins check the oxidation (formation), reduction (break down), and isomerization (rearrangement) of protein disulfide bonds via disulfide interchange activity. PDIs also have a chaperone activity by binding to misfolded proteins to prevent them from aggregating and targeting misfolded proteins for degradation [55].

Interestingly, treatment of P1 cells with resveratrol resulted in the decrease of P-SH content reflecting the resveratrol enhancement, in an AMPK-dependent manner, of the NAD^+^/NADH ratio [28], capable of restoring the basal level of CTR fibroblasts (Figure 6(c)).

Therefore, while the antioxidant effects of resveratrol are predominant in the CTR cells [22, 26] the capacity of this natural compound to modulate additional pathways is more evident in P1 cells [28, 51], including those regulating the glutathionylation status of proteins.

The redox state of thiol groups is related to glutathionylation of proteins which occurs in unstressed cells under physiological conditions as well as during cellular redox defense [56]. The glutathionylation is either a spontaneous or enzymatically driven finely controlled reversible process, which can involve both the GSH and GSSG [57]. To investigate the modifications in protein glutathionylated residues (PSSG), whole proteins were separated under nonreducing conditions. Western blotting analysis with an antibody against glutathionylated residues revealed, as expected, many protein bands (Figure 6(d)). Densitometric analysis showed a lower level of proteins detected by anti-GSH antibody in P1 cells compared to CTR cells (Figure 6(e)). Furthermore, resveratrol treatment resulted in a decrease of bands detected in control cells and, on the contrary, in an increase of bands detected in P1 cells (Figure 6(e)). These results (Figures 6(d) and 6(e)) are in agreement with the specific changes in P-SH levels (Figures 6(a), 6(b), and 6(c)), considering that a P-SH increase corresponds to a P-SSG decrease. Many enzymes are involved in the balance of the redox state of the SH groups, among which glutathione transferases (GST) catalyze protein glutathionylation [58]. Proteomic analysis reveals that resveratrol treatment of P1 cells leads to an increase of the glutathione S-transferase omega I (GSTO1) expression (Table 6), a protein possibly responsible for reversing the deregulation of GSH system and the redox state of protein sulfhydryl groups. In a *Drosophila* model of PD, upregulation of *Drosophila melanogaster* GST Sigma 1 (DmGSTO1) suppressed phenotypes caused by parkin loss of function, including the degeneration of DA neurons and muscle [59].

Deglutathionylation is catalyzed by thiol-disulfide oxidoreductase enzymes, such as glutaredoxin (GRX), thioredoxin (Trx), and protein disulfide isomerase (PDI). PRXs are also involved in the control of protein glutathionylation. Their primary role is associated to H_2_O_2_ detoxification, a process in which the active cysteines of PRX are oxidized. The recycling step of PRX involves the reduction of the disulfide bridge by the thioredoxin system, utilizing NADPH as a source of reducing power [60].

Overall, these data suggest that in P1 cells, there is a deregulation of GSH homeostasis and consequently of the redox state of sulfhydryl groups. The low availability of GSH and deregulation of protein folding processes in the ER, the first intracellular compartment for protein processing such as disulfide bond formation [61], could explain the high level of P-SH and the low level of glutathionylated protein observed P1 cells. In our previous study, proteomic analysis revealed a low level of PRDX4 [17], an ER-resident protein, in P1 compared to CTR cells. In the present study, a higher level of PRDX1, a cytosolic protein with antioxidant properties [62], was detected in P1 compared to CTR cells. Both peroxidases use thioredoxin as physiological reductant [48]. Resveratrol treatment restored GSH level and induced normal homeostasis of protein thiol groups in P1 cells. Moreover, in P1 cells, resveratrol treatment induced an upregulation of PRX1 and a downregulation of PRDX6, which uses glutathione as the physiological reductant, saving the amount of the glutathione for other activities.

### 3.4. Modulation of Chaperone Proteins upon Treatment with Resveratrol

Resveratrol, apart from being an effective scavenger of free radicals, may directly stimulate the cell defense against stress response through cellular chaperone in early time treatment [63]. Some members of the HSPs are differentially expressed in CTR and P1 samples after resveratrol treatment (Tables 5 and 6). Recently, many studies provided evidence that AMPK is a key mediator of the metabolic benefits produced by resveratrol, upstream of SIRT1 activation [64–67]. In the PD cellular model used in our previous study, we have shown that resveratrol regulates energy homeostasis through activation of AMPK and SIRT1 and raises mRNA expression of a number of PGC-1*α*'s target genes resulting in enhanced mitochondrial oxidative function, likely related to a decrease of oxidative stress and to an increase of mitochondrial biogenesis [28]. SIRT1 can deacetylate and activate heat-shock factor 1 (HSF1), which affects transcription of molecular chaperones [68].

In addition to protein refolding or degradation, HSPs also support a specialized autophagy mechanism called chaperone-mediated autophagy (CMA). This is a highly selective and constitutive subtype of autophagy that utilizes chaperone proteins and lysosomal receptors to directly target proteins into the lysosomal lumen for their degradation, under both physiological and pathological conditions to maintain cellular homeostasis [69–73]. There are multiple lines of evidence for the impairment of CMA activity in both familial and sporadic PD [74, 75]. In the CMA process, which is activated after macroautophagy and activation persists for days [76], the heat-shock cognate 70 (Hsc70/HSPA8), a constitutive chaperone, binds target proteins and transports them to the surface of endoplasmic reticulum where it specifically binds to lysosomal receptor protein LAMP-2A and HSP90, an inducible chaperone. Resveratrol treatment of P1 cells induces increased expression of both HSPA8 and HSP90, possibly leading to CMA. Interestingly, we have shown in our previous study that resveratrol treatment caused an enhanced macroautophagic flux through activation of an LC3-independent pathway [28].

Furthermore, concerning the behaviour of alpha-crystallin B chain (CRYAB), which belongs to the chaperone family whose main role is to bind improperly folded proteins to prevent protein aggregation [77], we have found that the treatment with resveratrol increased the expression of CRYAB in CRT cells and, on the contrary, induced a decreased expression in P1 cells, reestablishing the levels of control cells (Tables 5 and 7) (Figure 4).

HSPs are proving to be a therapeutic target in neurodegenerative disorders because the pathogenesis of these diseases is thought to be related to an abnormal increase of unfolded protein response (UPR), failure of UPS, and protein misfolding and/or aggregation [78] and their regulation could be mediated by polyphenols as resveratrol [79, 80].

### 3.5. Modulation of Metabolic Proteins upon Treatment with Resveratrol

As already described, resveratrol can carry out its functions by activating AMPK, a crucial cellular energy sensor. Once activated, it promotes ATP production by increasing the activity or expression of proteins involved in catabolism while conserving ATP by switching off biosynthetic pathways [81]. A significant number of proteins differentially expressed after resveratrol treatment are involved in energy metabolism pathways. We observed an upregulation of several proteins related to glycolysis in resveratrol-treated CTR cells. These include phosphoglycerate mutase 1 (PGAM1), triose phosphate isomerase (TPIS), and alpha enolase (ENOA). A changed rate of glycolysis may affect substrate levels for the tricarboxylic acid cycle and subsequent oxidative phosphorylation, in turn influencing ATP levels. Furthermore, we observed an upregulation of the cytoplasmic isoform of malate dehydrogenase (MDHc) in resveratrol-treated P1 cells. MDHc is a metabolic isoform involved in the malate-aspartate shuttle that aids in the transfer of reducing equivalents of NADH into the mitochondria. This is in line with the low steady-state cellular ratio NADH/NAD^+^ present in resveratrol-treated P1 cells, which indicates the enhancement of oxidative capacity attested by the increase in mitochondrial ATP production [28].

All these data confirm and extend our previous observations on the metabolic dysfunction of P1 fibroblasts, which show a deregulation of pathways involved in key cellular processes such as protein folding, protein degradation, and cellular redox balance. The analysis of differentially expressed proteins identified after resveratrol treatment of CTR and P1 cells reveals the great ability of resveratrol to act on protein expression modifying pathway and reversing the molecular defects in P1 fibroblast cells.

## 4. Conclusions

P1 fibroblasts are characterized by a dysregulated expression of proteins linked to biological processes regarding cell movement or subcellular component, assembly and protein folding, calcium ion binding, unfolded protein binding, and redox homeostasis. In this study, we show the biological effects of resveratrol acting through the modulation of the expression of proteins involved in protecting cells from the damaging effects of ROS, in protein refolding or degradation, and, specifically, in chaperone-mediated autophagy.

Overall, the complex proteome alterations shown in this ex vivo model of PD could provide further insights into the pathogenic processes involved in the disease. Importantly, the elucidation of the biomarkers might provide new therapeutic targets for the treatment and prevention of PD. Evidences are emerging to support the potential of small bioactive molecules, as resveratrol, against neurodegenerative disorders, to control and modulate ROS and abnormal protein.

## Figures and Tables

**Table 1:** List of differentially expressed proteins identified by MS/MS in CTR and P1 samples.

Spot number	Swiss-Prot accession number	Protein name	Gene name	Mascot score		Sequence coverage MS	Sequence coverage MS/MS	Peptides	Fold change P1/CTR	*p* value	Instrument
Spot 1	Q9Y4L1	Hypoxia-upregulated protein (HYOU1)	*HYOU1*	181	106	25%	2%	K.LCQGLFFR.V K.QADNPHVALYQAR.F	2.2	^∗∗∗^	MALDI-TOF/TOF
Spot 2	Q06830	Peroxiredoxin-1 (PRDX1)	*PRDX1*	/	47	/	29%	R.TIAQDYGVLK.A K.ATAVMPDGQFK.D + oxidation (M) R.LVQAFQFTDK.H R.QITVNDLPVGR.S	1.6	^∗∗∗^	ESI-Trap
Spot 3	P09936	Ubiquitin carboxyl-terminal hydrolase isozyme L1 (UCHL1)	*UCHL1*	86	220	49%	19%	R.LGVAGQWR.F R.MPFPVNHGASSEDTLLK.D + oxidation (M) K.NEAIQAAHDAVAQEGQCR.V	3.8	^∗∗∗^	MALDI-TOF/TOF
Spot 4	P63244	Guanine nucleotide-binding protein subunit beta-2-like 1 (GBLP)	*GNB2L1*	97	84	38%	6%	R.VWQVTIGTR.-R.DETNYGIPQR.A	2.3	^∗∗^	MALDI-TOF/TOF
Spot 5	Q8WUM4	Programmed cell death 6 interacting protein (PDC6I)	*PDCD6IP*	175	167	29%	4%	R.TPSNELYKPLR.A R.YYDQICSSIEPK.F K.MVPVSVQQSLAAYNQR.K	1.6	^∗^	MALDI-TOF/TOF
Spot 6	P15311	Ezrin (EZRI)	*EZR*	111	134	25%	4%	K.IGFPWSEIR.N R.IQVWHAEHR.G K.KAPDFVFYAPR.L	−5.5	^∗∗∗^	MALDI-TOF/TOF
Spot 7	P17987	T-complex protein 1 subunit alpha (TCPA)	*TCP1*	96	214	22%	6%	K.YFVEAGAMAVR.R K.IHPTSVISGYR.L R.YINENLIVNTDELGR.D	−1.6	^∗∗∗^	MALDI-TOF/TOF
Spot 8	P34897	Serine hydroxymethyltransferase mitochondrial (GLYM)	*SHMT2*	61	142	27%	9%	R.LIIAGTSAYAR.L K.TGLIDYNQLALTAR.L R.GYSLVSGGTDNHLVLVDLRPK.G	1.8	^∗∗∗^	MALDI-TOF/TOF
Spot 9	Q16658	Fascin (FSCN1)	*FSCN1*	150	171	42%	6%	R.FLIVAHDDGR.W K.LINRPIIVFR.G K.NASCYFDIEWR.D	−1.7	^∗∗^	MALDI-TOF/TOF
Spot 10	P49368	T-complex protein 1 subunit gamma (TCPG)	*CCT3*	87	145	12%	6%	R.NLQDAMQVCR.N R.TLIQNCGASTIR.L K.AMTGVEQWPYR.A + oxidation (M)	−1.8	^∗∗^	MALDI-TOF/TOF
Spot 11	P07741	Adenine phosphoribosyltransferase (APT)	*APRT*	60	147	28%	21%	R.IDYIAGLDSR.G R.SFPDFPTPGVVFR.D K.AELEIQKDALEPGQR.V	1.6	^∗∗^	MALDI-TOF/TOF
Spot 12	P07195	L-lactate dehydrogenase B chain (LDH-B)	*LDHB*	125	127	33%	11%	K.IVVVTAGVR.G R.VIGSGCNLDSAR.F K.GEMMDLQHGSLFLQTPK.I + oxidation (M)	1.9	^∗∗∗^	MALDI-TOF/TOF
Spot 13	P35998	26S proteasome regulatory subunit 7 (PRS7)	*PSMC2*	61	67	20%	9%	R.KIEFSLPDLEGR.T K.QTLQSEQPLVAR.C K.ACLIFFDEIDAIGGAR.F	1.8	^∗∗^	MALDI-TOF/TOF
Spot 14	P07355	Annexin A2 (ANXA2)	*ANXA2*	67	82	21%	7%	K.WISIMTER.S + oxidation (M) K.AYTNFDAER.D R.QDIAFAYQR.R	2	^∗∗∗^	MALDI-TOF/TOF
Spot 15	P00338	L-lactate dehydrogenase A chain (LDHA)	*LDHA*	96	83	26%	7%	K.LVIITAGAR.Q K.DQLIYNLLKEEQTPQNK.I	2	^∗∗∗^	MALDI-TOF/TOF

Spot numbers match those reported in the representative 2-DE images shown in Figure 1. Accession number in Swiss-Prot/UniprotKB (http://www.uniprot.org/). Fold change (P1 versus CTR cells) was calculated dividing the average of %V P1 cells by the average of %V CTR cells of three independent experiments. *t*-test was performed by GraphPad v4.0 software to determine if the relative change was statistically significant (*p* < 0.05); ^∗^*p* < 0.05; ^∗∗^*p* < 0.01; ^∗∗∗^*p* < 0.001.

**Table 2:** List of significantly enriched biological processes in CTR versus P1 protein dataset identified by STRING software.

Biological process (GO)			
Pathway ID	Pathway description	Count in gene set	False discovery rate
GO:0006928	Movement of a cell or subcellular component	16	1.1*e*−05
GO:0006457	Protein folding	8	4.08*e*−05
GO:0030049	Muscle filament sliding	5	4.08*e*−05
GO:0006986	Response to unfolded protein	7	5.67*e*−05
GO:0022607	Cellular component assembly	16	9.99*e*−05

**Table 3:** List of significantly enriched molecular functions in CTR versus P1 protein dataset identified by STRING software.

Molecular function (GO)			
Pathway ID	Pathway description	Count in gene set	False discovery rate
GO:0005515	Protein binding	30	1.16*e*−08
GO:0005509	Calcium ion binding	12	7.71*e*−06
GO:0051082	Unfolded protein binding	6	7.71*e*−06
GO:0003723	RNA binding	16	1.33*e*−05
GO:0044822	Poly(A) RNA binding	13	0.000172

**Table 4:** List of significantly enriched molecular functions in CTR versus P1 protein dataset identified by STRING software.

KEGG pathways (GO)			
Pathway ID	Pathway description	Count in gene set	False discovery rate
04141	Protein processing in endoplasmic reticulum	7	1.25*e*−05

**Table 5:** List of differentially expressed proteins identified by MS/MS in CTR and CTR-Res-treated samples.

Spot number	Swiss-Prot accession number	Protein name	Gene name	Mascot score		Sequence coverage MS	Sequence coverage MS/MS	Peptides	Fold change CTR-Res/CTR	*p* value	Instrument
				PMF	MS/MS						
Spot 1	P41250	Glicine-tRNA ligase (SYG)	*GARS*	91	144	21%	6%	K.NNIIQTWR.Q R.SCYDLSCHAR.A K.LPFAAAQIGNSFR.N K.TSYGWIEIVGCCADR.S	−1.6	^∗∗^	MALDI-TOF/TOF
Spot 2	Q9Y4L1	Hypoxia upregulated protein (HYOU1)	*HYOU1*	181	106	25%	2%	K.LCQGLFFR.V K.QADNPHVALYQAR.F	−2.3	^∗∗∗^	MALDI-TOF/TOF
Spot 3	O43707	Alpha-actinin 4 (ACTN4)	*ACTN4*	130	111	26%	3%	K.GYEEWLLNEIR.R R.ASFNHFDKDHGGALGPEEFK.A	−1.9	^∗∗∗^	MALDI-TOF/TOF
Spot 4	P55072	Transitional endoplasmic reticulum ATPase (TERA)	*VCP*	/	97	/	24%	R.GILLYGPPGTGK.T R.EVDIGIPDATGR.L R.WALSQSNPSALR.E R.IVSQLLTLMDGLK.Q R.LGDVISIQPCPDVK.Y R.LDQLIYIPLPDEK.S K.MTNGFSGADLTEICQR.A K.NAPAIIFIDELDAIAPK.R K.MTNGFSGADLTEICQR.A + oxidation (M) R.QAAPCVLFFDELDSIAK.A R.EDEEESLNEVGYDDIGGCR.K R.ETVVEVPQVTWEDIGGLEDVKR.E	−2.5	^∗∗^	ESI-Trap
Spot 5	P18669	Phosphoglycerate mutase 1(PGAM1)	*PGAM1*	73	149	27%	8%	R.VLIAAHGNSLR.G R.HGESSAWNLENR.F	1.7	^∗^	**MALDI-TOF/TOF**
Spot 6	P60174	Triosephosphate isomerase (TPIS)	*TPI1*	112	175	42%	9%	R.HVFGESDELIGQK.V K.DCGATWVVLGHSER.R	1.6	^∗∗^	MALDI-TOF/TOF
Spot 7	Q06830	Peroxiredoxin-1 (PRDX1)	*PRDX1*	/	47	/	29%	R.TIAQDYGVLK.A K.ATAVMPDGQFK.D + oxidation (M) R.LVQAFQFTDK.H R.QITVNDLPVGR.S	−1.9	^∗∗∗^	ESI-Trap
Spot 8	P06733	Alpha enolase (ENOA)	*ENO1*	145	162	51%	14%	R.YISPDQLADLYK,S K.LAQANGWGVMVSHR.S + oxidation (M) R.AAVPSGASTGIYEALELR.D K.LAMQEFMILPVGAANFR.E + 2 oxidation (M)	2.2	^∗∗^	MALDI-TOF/TOF
Spot 9	P06733	Alpha enolase (ENOA)	*ENO1*	145	96	51%	8%	R.AAVPSGASTGIYEALELR.D K.LAMQEFMILPVGAANFR.E + 2 oxidation (M)	2.4	^∗∗∗^	MALDI-TOF/TOF
Spot 10	P02511	Alpha-crystallin B chain (CRYAB)	*CRYAB*	103	79	12%	11%	R.QDEHGFISR.E R.RPFFPFHSPSR.L	1.6	^∗^	MALDI-TOF/TOF
Spot 11	Q99497	DJ-1 (PARK7)	*PARK7*	88	44	50%	19%	K.DGLILTSR.G K.MMNGGHYTYSENR.V + oxidation (M) K.GAEEMETVIPVDVMR.R	1.7	^∗^	MALDI-TOF/TOF
Spot 12	Q14764	Major vault protein (MVP)	MVP	160	117	31%	3%	R.GAVASVTFDDFHK.N R.IPPYHYIHVLDGNSNVSR.V	−1.6	^∗∗^	MALDI-TOF/TOF
Spot 13	P02545	Prelamin A/C (LMNA)	*LMNA*	130	185	29%	5%	R.LADALQELR.A R.TLEGELHDLR.G R.NSNLVGAAHEELQQSR.I	1.8	^∗∗^	MALDI-TOF/TOF
Spot 14	P30101	Protein disulfide-isomerase A3 (PDIA3)	*PDIA3*	107	59	23%	1%	K.QAGPASVPLR.T	−2.1	^∗∗∗^	MALDI-TOF/TOF
Spot 15	P30086	Phosphatidylethanolamine-binding protein 1 (PEBP1)	*PEBP1*	187	238	67%	22%	K.LYTLVLTDPDAPSR.K K.NRPTSISWDGLDSGK.L R.YVWLVYEQDRPLK.C	2.2	^∗∗∗^	MALDI-TOF/TOF
Spot 16	P32119	Peroxiredoxin-2 (PRDX2)	*PRDX2*	93	324	47%	14%	R.QITVNDLPVGR.S K.EGGLGPLNIPLLADVTR.R R.KEGGLGPLNIPLLADVTR.R	1.8	^∗∗^	MALDI-TOF/TOF
Spot 18	P08670	Vimentin (VIME)	*VIM*	62	124	15%	13%	K.FADLSEAANRNNDALR.Q R.QVQSLTCEVDALKGTNESLER.Q	−1.6	^∗∗∗^	MALDI-TOF/TOF
Spot 19	P07237	Protein disulfide-isomerase (PDIA1)	*P4HB*	130	142	27%	7%	K.FFPASADR.T K.VDATEESDLAQQYGVR.G K.ILFIFIDSDHTDNQR.I	−2	^∗∗∗^	MALDI-TOF/TOF
Spot 20	P62826	GTP-binding nuclear protein Ran	*RAN*	74	99	25%	8%	K.YVATLGVEVHPLVFHTNR.G K.KYVATLGVEVHPLVFHTNR.G	1.8	^∗∗^	MALDI-TOF/TOF

Spot numbers match those reported in the representative 2-DE images shown in Figure 2. Accession number in Swiss-Prot/UniprotKB (http://www.uniprot.org/). Fold change (resveratrol-treated cells versus control cells) was calculated dividing the average of %V resveratrol-treated cells by the average of %V control cells of three independent experiments. *t*-test was performed by GraphPad v4.0 software to determine if the relative change was statistically significant (*p* < 0.05); ^∗^*p* < 0.05; ^∗∗^*p* < 0.01; ^∗∗∗^*p* < 0.001.

**Table 6:** List of differentially expressed proteins identified by MS/MS in P1 and P1-Res-treated samples.

Spot number	Swiss-Prot accession number	Protein name	Gene name	Mascot score		Sequence coverage MS	Sequence coverage MS/MS	Peptides	Fold change P1 Res/P1	*p* value	Instrument
				PMF	MS/MS						
Spot 21	Q14697	Neutral alpha-glucosidase AB (GANAB)	*GANAB*	265	137	37%	3%	K.AEKDEPGAWEETFK.T K.MMDYLQGSGETPQTDVR.W + oxidation (M)	1.9	^∗∗^	MALDI-TOF/TOF
Spot 22	P18206	Vinculin (VINC)	*VCL*	/	105	/	10%	R.WIDNPTVDDR.G R.VMLVNSMNTVK.E + oxidation (M) K.MTGLVDEAIDTK.S + oxidation (M) K.QVATALQNLQTK.T K.AQQVSQGLDVLTAK.V R.DPSASPGDAGEQAIR.Q R.GILSGTSDLLLTFDEAEVR.K R.GILSGTSDLLLTFDEAEVR.K K.LLAVAATAPPDAPNREEVFDER.A	1.6	^∗^	Esi-Trap
Spot 23	P42224	STAT1 (STAT1)	*STAT1*	86	85	24%	4%	K.YLYPNIDKDHAFGK.Y K.QDWEHAANDVSFATIR.F	2.2	^∗∗∗^	MALDI-TOF/TOF
Spot 24	P02511	Alpha-crystallin B chain (CRYAB)	*CRYAB*	103	79	12%	11%	R.QDEHGFISR.E R.RPFFPFHSPSR.L	−2.3	^∗∗∗^	MALDI-TOF/TOF
Spot 25	P63244	Guanine nucleotide-binding protein subunit beta-2-like 1 (GBLP)	*GNB2L1*	97	84	38%	6%	R.VWQVTIGTR.-R.DETNYGIPQR.A	−1.9	^∗∗^	MALDI-TOF/TOF
Spot 26	P61163	Alpha-centractin (ACTZ)	*ACTR1A*	78	87	23%	4%	K.AQYYLPDGSTIEIGPSR.F	−1.6	^∗^	MALDI-TOF/TOF
Spot 27	P61160	Actin-related protein 2 (ARP2)	*ACTR2*	61	151	20%	10%	K.HLWDYTFGPEK.L R.GYAFNHSADFETVR.M K.HIVLSGGSTMYPGLPSR.L + oxidation (M)	−1.7	^∗∗^	MALDI-TOF/TOF
Spot 28	P00367/Q15654	Glutamate dehydrogenase 1, mitochondrial (DHE3)/thyroid receptor-interacting protein 6 (TRIP6)	*GLUD1/TRIP6*	84/123	91/73	29%/39%	3%/8%	K.MVEGFFDR.G R.DDGSWEVIEGYR.A / R.GTPGPPPAHGAALQPHPR.V R.VNFCPLPSEQCYQAPGGPEDR.G	−2.1	^∗∗^	MALDI-TOF/TOF
Spot 29	Q06830	Peroxiredoxin-1 (PRDX1)	*PRDX1*	/	47	/	29%	R.TIAQDYGVLK.A K.ATAVMPDGQFK.D + oxidation (M) R.LVQAFQFTDK.H R.QITVNDLPVGR.S	2	^∗∗∗^	ESI-Trap
Spot 30	P52907	F-actin-capping protein subunit alpha-1 (CAZA1)	*CAPZA1*	59	109	34%	9%	R.LLLNNDNLLR.E K.FITHAPPGEFNEVFNDVR.L	1.8	^∗∗^	MALDI-TOF/TOF
Spot 31	P40925	Malate dehydrogenase, cytoplasmic (MDHC)	*MDH1*	58	173	18%	6%	K.GEFVTTVQQR.G K.FVEGLPINDFSR.E	1.7	^∗∗^	MALDI-TOF/TOF
Spot 33	P78417	Glutathione S-transferase omega I (GSTO1)	*GSTO1*	102	137	36%	17%	R.FCPFAER.T R.HEVININLK.N K.GSAPPGPVPEGSIR.I K.EDYAGLKEEFR.K	−1.6	^∗∗∗^	MALDI-TOF/TOF
Spot 34	P09960	Leukotriene A-4 hydrolase (LKHA4)	*LTA4H*	66	42	17%	5%	R.TLTGTAALTVQSQEDNLR.S R.MQEVYNFAINNSEIR.F + oxidation (M)	−1.6	^∗^	MALDI- TOF/TOF
Spot 35	P07900	Heat-shock protein HSP 90-alpha (HS90A)	*HSP90AA1*	/	70	/	12%	K.DQVANSAFVER.L K.ADLINNLGTIAK.S R.ELISNSSDALDK.I K.EDQTEYLEER.R K.EGLELPEDEEEK.K R.GVVDSEDLPLNISR.E R.NPDDITNEEYGEFYK.S K.TKPIWTRNPDDITNEEYGEFYK.S	−1.5	^∗^	Esi-Trap
Spot 37	P30086	Phosphatidylethanolamine-binding protein 1 (PEBP1)	*PEBP1*	61	177	28%	22%	K.LYTLVLTDPDAPSR.K K.NRPTSISWDGLDSGK.L R.YVWLVYEQDRPLK.C	2	^∗∗∗^	MALDI- TOF/TOF
Spot 39	P61160	Actin-related protein 2 (ARP2)	*ACTR2*	61	151	20%	10%	K.HLWDYTFGPEK.L R.GYAFNHSADFETVR.M K.HIVLSGGSTMYPGLPSR.L + oxidation (M)	1.7	^∗∗^	MALDI- TOF/TOF
Spot 41	P30086	Phosphatidylethanolamine-binding protein 1 (PEBP1)	*PEBP1*	187	238	67%	22%	K.LYTLVLTDPDAPSR.K K.NRPTSISWDGLDSGK.L R.YVWLVYEQDRPLK.C	−1.6	^∗∗^	MALDI-TOF/TOF
Spot 42	P13010	X-ray repair cross-complementing protein 5 (XRCC5)	*XRCC5*	89	110	12%	4%	R.LFQCLLHR.A R.HIEIFTDLSSR.F R.ANPQVAFPHIK.H	1.8	^∗∗∗^	MALDI-TOF/TOF
Spot 44	P30041	Peroxiredoxin-6 (PRDX6)	*PRDX6*	122	244	61%	16.5%	R.NFDEILR.V K.LPFPIIDDR.N M.PGGLLLGDVAPNFEANTTVGR.I	−1.6	^∗∗^	MALDI-TOF/TOF
Spot 45	P11142	Heat-shock cognate 71 kDa protein (HSP7C)	*HSPA8*	/	178	/	28%	K.VEIIANDQGNR.T R.FEELNADLFR.G R.FDDAVVQSDMK.H K.NSLESYAFNMK.A K.CNEIINWLDK.N K.NSLESYAFNMK.A + oxidation (M) K.SQIHDIVLVGGSTR.I R.TTPSYVAFTDTER.L K.SFYPEEVSSMVLTK.M K.NQVAMNPTNTVFDAK.R R.IINEPTAAAIAYGLDK.K K.STAGDTHLGGEDFDNR.M K.TVTNAVVTVPAYFNDSQR.Q K.TVTNAVVTVPAYFNDSQR.Q K.SINPDEAVAYGAAVQAAILSGDK.S	2.1	^∗∗∗^	MALDI-TOF/TOF
Spot 46	P04792	Heat-shock protein beta 1 (HSPB1)	*HSPB1*	127	171	51%	13%	R.LFDQAFGLPR.L K.LATQSNEITIPVTFESR.A	1.6	^∗∗^	MALDI-TOF/TOF
Spot 47	P09936	Ubiquitin carboxyl-terminal hydrolase isozyme L1 (UCHL1)	*UCHL1*	86	220	49%	19%	R.LGVAGQWR.F R.MPFPVNHGASSEDTLLK.D + oxidation (M) K.NEAIQAAHDAVAQEGQCR.V	1.7	^∗∗^	MALDI-TOF/TOF
Spot 48	P15311	Ezrin (EZRI)	*EZR*	111	134	25%	4%	K.IGFPWSEIR.N R.IQVWHAEHR.G K.KAPDFVFYAPR.L	−1.7	^∗∗^	MALDI-TOF/TOF
Spot 49	P29401	Transketolase (TKT)	*TKT*	139	222	25%	7%	R.VLDPFTIKPLDR.K R.TVPFCSTFAAFFTR.A R.FIECYIAEQNMVSIAVGCATR.N	−1.5	^∗∗^	MALDI-TOF/TOF
Spot 50	P42704	Leucine-rich PPR motif-containing protein, mitochondrial (LPPRC)	*LRPPRC*	109	37	15%	1%	K.SNTLPISLQSIR.S K.VIEPQYFGLAYLFR.K	−2.1	^∗∗∗^	MALDI-TOF/TOF
Spot 51	P23526	Adenosylhomocysteinase (SAHH)	*AHCY*	/	84	/	10%	K.VPAINVNDSVTK.S K.ALDIAENEMPGLMR.M + oxidation (M) K.ALDIAENEMPGLMR.M + 2 oxidation (M) R.ATDVMIAGKVAVVAGYGDVGK.G + oxidation (M)	−1.7	^∗∗^	Esi-Trap
Spot 52	P07954	Fumaratehydratase, mitochondrial (FUMH)	*FH*	66	158	18%	5%	R.IEYDTFGELK.V R.IYELAAGGTAVGTGLNTR.I	−1.9	^∗∗∗^	MALDI-TOF/TOF

Spot numbers match those reported in the representative 2-DE images shown in Figure 3. Accession number in Swiss-Prot/UniprotKB (http://www.uniprot.org/). Fold change (P1 resveratrol-treated cells versus P1 cells) was calculated dividing the average of %V P1 resveratrol-treated cells by the average of %V P1 cells of three independent experiments. *t*-test was performed by GraphPad v4.0 software to determine if the relative change was statistically significant (*p* < 0.05); ^∗^*p* < 0.05; ^∗∗^*p* < 0.01; ^∗∗∗^*p* < 0.001.

**Table 7:** List of significantly enriched biological processes in CTR versus CTR-Res protein dataset identified by STRING software.

Biological process (GO)			
Pathway ID	Pathway description	Count in gene set	False discovery rate
GO:0001666	Response to hypoxia	5	0.00305
GO:0034976	Response to endoplasmic reticulum stress	5	0.00305
GO:0042743	Hydrogen peroxide metabolic process	3	0.00305
GO:0061621	Canonical glycolysis	3	0.00305
GO:2000152	Regulation of ubiquitin-specific protease activity	2	0.00305

**Table 8:** List of significantly enriched molecular functions in CTR versus CTR-Res protein dataset identified by STRING software.

Molecular function (GO)			
Pathway ID	Pathway description	Count in gene set	False discovery rate
GO:0051920	Peroxiredoxin activity	3	8.22*e*−05
GO:0003723	RNA binding	10	0.000179
GO:0044822	Poly(A) RNA binding	9	0.000179
GO:0005515	Protein binding	13	0.00361
GO:0008379	Thioredoxin peroxidase activity	2	0.00361

**Table 9:** List of significantly enriched molecular functions in P1 versus P1-Res protein dataset identified by STRING software.

Molecular function (GO)			
Pathway ID	Pathway description	Count in gene set	False discovery rate
GO:0019899	Enzyme binding	11	0.000172
GO:0031625	Ubiquitin protein ligase binding	6	0.000172
GO:0044822	Poly(A) RNA binding	10	0.000228
GO:0005515	Protein binding	15	0.00801
GO:0051920	Peroxiredoxin activity	2	0.0162
